# Point-of-Care Thoracic Ultrasonography in Patients With Cirrhosis and Liver Failure

**DOI:** 10.7759/cureus.15559

**Published:** 2021-06-10

**Authors:** Kamal Kajal, Madhumita Premkumar, Sreedhara B Chaluvashetty, Harish Bhujade, Anand V Kulkarni

**Affiliations:** 1 Anesthesia and Critical Care, Post Graduate Institute of Medical Education & Research, Chandigarh, IND; 2 Hepatology, Post Graduate Institute of Medical Education & Research, Chandigarh, IND; 3 Radiodiagnosis, Post Graduate Institute of Medical Education & Research, Chandigarh, IND; 4 Hepatology, Asian Institute of Gastroenterology, Hyderabad, IND

**Keywords:** lung ultrasound, point-of-care, pocus, cirrhosis, intensive care, pneumonia, liver transplantation

## Abstract

Point-of-care ultrasonography (POCUS) helps determine liver-related pathologies like an abscess, portal vein or hepatic vein thromboses, presence of ascites, site for pleural or ascitic paracentesis, and guiding biopsies. POCUS is revolutionizing the management of critically ill patients presenting with pneumonia, acute respiratory distress syndrome, acute-on-chronic liver failure, and in the emergency. The objectives of thoracic ultrasonography (TUS) are to aid the clinician in differentiating between pneumonia, effusions, interstitial edema and collections, and in estimating the volume status of patients with liver disease using inferior vena cava dynamic indices. The use of POCUS in patients with cirrhosis has since evolved. It is now widely used to help diagnose volume status, left ventricular diastolic dysfunction, myocardial infarction, and right ventricular dilation due to pulmonary embolism and to determine the causes for weaning failures such as effusions, lung collapse, and pneumothorax. During the Coronavirus Disease 2019 (COVID-19) pandemic, moving patients for computed tomography can be difficult. Therefore, TUS is now essential in liver transplantation and intensive care practice to assess ventilatory pressures, cardiac function, and fluid management. This review indicates the current and optimized use of TUS, offers a practical guide on TUS in the liver intensive care unit (ICU), and presents a diagnostic pathway for determining lung and pleural pathology, resolution of respiratory failure, and aid weaning from mechanical ventilation.

## Introduction and background

Thoracic ultrasonography (TUS) of the lung and pleura is a key component of critical care point of care ultrasonography (POCUS) in patients with cirrhosis, acute-on-chronic liver failure (ACLF), and those with hepatic hydrothorax [1]. In critically ill patients with cirrhosis, TUS can be used to diagnose patients with undifferentiated dyspnea [2], suspected pneumothorax [3], hemothorax, pneumonia [4,5], lung collapse [6], ventilator-associated pneumonia [7], titration of positive end-expiratory pressure in acute respiratory distress syndrome (ARDS) [6,7], weaning failure [8,9], and pulmonary embolism [10]. POCUS can be performed by clinicians using inexpensive handheld machines, whereas consultative ultrasonography is performed by radiologists in a suite with expensive and advanced ultrasound machines. The main indications for performing a combination of POCUS, TUS, and transthoracic echocardiography (TTE) are to improve bedside diagnosis of pulmonary diseases and hemodynamic status in patients with liver disease which facilitates clinical decision pathways in intensive care units (ICUs) [8], operating rooms, or emergency department (Table 1) [2-11].

**Table 1:** Indications for thoracic ultrasonography (TUS) in patients with cirrhosis [2-11] Abbreviations: ARDS, acute respiratory distress syndrome; TUS, thoracic ultrasonography; IVC, inferior vena cava; LUS, lung ultrasound

Condition	Differential	Clinical Decision Making
Chest pain [4]	Myocardial infarction, myocarditis, pulmonary embolism	Cardiac comorbidities are well described in cirrhosis and need definitive treatment
Dyspnea [2]	Pneumonia, lung collapse, pneumothorax, hepato-pulmonary syndrome	Undifferentiated dyspnea can be diagnosed using TUS. Hepatopulmonary syndrome (HPS) requires screening using saline contrast echocardiography which can be done at the bedside with a phased array probe.
Hypoxia	Pneumonia, acute respiratory distress syndrome (ARDS)	Relevant in patients with liver disease with sepsis and pancreatitis
Shock	Dehydration, over diuresis, myocardial infarction	Determine volume resuscitation in cirrhosis using inferior vena cava (IVC) collapsibility and rule out cardiogenic causes. The use of central venous pressure (CVP) in cirrhosis is less reliable for guiding fluids in critical illness.
Pleurisy	Pulmonary embolism, pneumothorax, post-biopsy pain	Pleuritic pain can be due to acute pulmonary embolism. Since cirrhosis is a procoagulant state, pulmonary embolism is not uncommon in critically ill patients with cirrhosis.
Thoracic trauma	Hemothorax	Blunt trauma or road traffic accidents
ARDS [2,6]	Interstitial edema, collapse of lung, pancreatitis	Respiratory failure diagnosis, ventilatory settings modification. Differentiation between pneumonia, lung collapse, and effusion using LUS is useful to guide ventilatory settings in cirrhosis and acute-on-chronic liver failure (ACLF)
Pulmonary thromboembolism [9]	Pneumonia	Anticoagulation can be started at the bedside in case of the diagnostic right-atrial/right-ventricular collapse seen on bedside echocardiography.
Identify right main stem bronchus intubation	The selective intubation of a lung segment can be assessed	Tube placement in mechanical ventilation
Ultrasound-guided interventions [10]	Central line placement	Pneumothorax/hemothorax after line placement
	Biopsy	A liver biopsy can be done at the bedside. Ultrasound-guided fine-needle aspiration can be done from suspicious lesions.
	Thoracentesis	Diagnostic or Therapeutic thoracentesis can be done with percutaneous drain placement at the bedside.
	Drainage of amebic liver abscess	Single time aspiration or drain placement

This is now of paramount importance in the Coronavirus Disease 2019 (COVID-19) era, as moving patients to radiology units for computed tomography or radiographs is difficult and more clinicians, including hepatologists and gastroenterologists, should learn the skill of a fast POCUS of the lung, heart, and abdomen [12]. There are restrictions on the movement of healthcare personnel to ICUs across all specialties. We have already reported the effect of COVID-19 on liver biochemistries [13]. Now, it is increasingly important that ICUs and liver transplantation units become self-reliant in bedside diagnosis of pulmonary pathology by performing a simple TUS. The use of ‘telemedicine’ tools can also aid expert radiologists in reviewing the real-time images obtained by the primary physician in the ICU. The POCUS performed at the bedside can be interpreted remotely by an experienced radiologist; this has enabled early and appropriate decisions, especially in COVID-19 units. The concept is now adopted across other ICUs to minimize healthcare worker and patient movement [14].

## Review

Equipment required for TUS in patients with liver disease

A wide variety of scanning equipment can be used for bedside pleural ultrasonography and associated procedures in patients with cirrhosis and liver failure. In patients with liver disease, modification of scanning technique needs to be done due to the presence of ascites and limited scanning window. In such critically ill cirrhotic patients, the use of TUS can guide critical care decisions such as end points of fluid resuscitation, lung recruitment maneuvers, adjustment of pressure settings on mechanical ventilation, monitoring pneumonia, and detection of pneumothorax [9,10].

Transducers

The TUS is best done using either the curvilinear transducer (1-5 MHz), which is useful for imaging the base of the lung, costophrenic angles, pleural surface, and lower intercostal spaces as it has a large footprint. Once the pathology is identified, the clinician can switch to a linear probe. This high-frequency probe can image up to a depth of 4 cm with high resolution and is used to see the A-lines and B-lines. However, only a small imaging footprint is available, albeit in high resolution. Therefore a 1-3 MHz phased array probe can also be used for TUS and can be used to scan with ease in the intercostal spaces. The phased array probe is also used for complimentary echocardiography done at the bedside. The phased array probe is useful for imaging more diffuse lung and pleural pathology. This probe also has enough tissue penetration depth to image structures deep within the thorax, unlike the linear high-frequency probe. This is the best probe for assessing lung sliding, identification of pleural lines, and procedural guidance [15,16].

POCUS is now accepted in critical care practice worldwide and has gained increased acceptance in hepatology and liver transplantation practice. Usually, we begin with a POCUS of the abdomen and move to the lung bases with the curvilinear probe with an abdominal preset. The lung is scanned using the abdominal preset, but the lung preset should be used for anterior views. The abdominal preset is useful for imaging solid lesions like consolidation. A depth of 9-14 cm should be chosen for anterior views. Then, the transducer is switched to the linear probe with a lung preset, and the lung sliding is assessed and areas of interest identified. The lung ultrasound (LUS) score is calculated using the mapping technique described in a subsequent section. Lastly, we switch to a phased array probe to visualize specific areas like lung parenchyma and deeper structures. The phased array probe is then switched to a cardiac preset, and basic echocardiography is done to complete the POCUS examination relevant to the patients with a critical illness. 

Gain or depth may need to be decreased to appreciate the hyperechoic pleural line clearly. We are required to view the intraabdominal organs (liver or spleen) and the diaphragmatic pleural reflection in dependent views. The pleura should be imaged by keeping the probe perpendicular to it. The operator should assess for lung sliding, presence of A- and B-lines, evidence of pleural effusion, consolidation of the underlying lung, and associated infra-diaphragmatic structures like the liver and the spleen in patients with liver disease.

Table 2 shows the normal findings and comparison of TUS with conventional computed tomography (CT) and radiographs [15-20].

**Table 2:** TUS in liver disease, differentials, classical findings, and comparison with chest computed tomography (CT) and chest radiographs (CXR) Abbreviations: ARDS, acute respiratory distress syndrome; CXR, chest radiograph; CT, computed tomography; COPD, chronic obstructive pulmonary disease; RV, right ventricle; PAOP, pulmonary artery occlusion pressure; PEEP, peak end expiratory pressure; TTE, transthoracic echocardiography; TUS, thoracic ultrasound; LUS, lung ultrasound; TR, tricuspid regurgitation

Condition	Findings on TUS	Ultrasound diagnosis	Comparison with CT/ Radiograph	Advantages	Differentials
Normal	An A-line pattern with lung sliding indicates a normal aeration pattern	Lung sliding present with A-lines/ less than two isolated B-lines	Bedside diagnosis	More cost-efficient. No ionizing radiation	Lung sliding is also seen in breath-holding, apnea in endotracheal tube displacement, or pleurodesis.
Pneumothorax [14]	Loss of lung sliding	Loss of lung sliding can also be seen with pleurodesis or breath-holding	High PEEP, COPD/emphysema, mainstem intubation, lung bullae, and ventilator apnea may also lead to loss of lung sliding	Bedside drainage	Presence of B-lines and/or pleural pulse (transmitted pleural movement due to the heart) also rules out pneumothorax
Pleural effusion/ Hepatic hydrothorax	Anechoic area surrounded by typical anatomic boundaries	Fluid in pleural cavity	Sensitivity 93% versus 47% with CXR	TUS is better for checking pleural effusion septations, differentiating pleural fluid from chest wall tumor invasion, pleural thickening, and pleural masses compared with chest CT scan	Empyema, hemothorax, pleural mass
Loculated pleural effusion	Anechoic area with septations	Loculated fluid in pleural cavity; suggests chronic collection	The sensitivity of diagnosing a complicated parapneumonic effusion using LUS, chest radiography, and CT was 69%, 61%, and 76%, respectively.	Pleural effusions can also loculate because of adhesions.	Hemothorax, pyothorax, chylothorax, or tuberculous pleuritis
Pulmonary embolism [11]	Peripheral wedge-shaped abnormalities or alternate etiologies (e.g., alveolar consolidation)	RV dilation, ventricle size ratio, abnormal septal motion, TR, RV hypokinesis, pulmonary hypertension, RV end-diastolic diameter	McConnel Sign (RV dysfunction with characteristic sparing of the apex)	Early anticoagulation can be offered.	Pneumonia, lung collapse
Pneumonia [15]	Subpleural consolidations and dynamic air bronchograms	Consolidation	Sensitivity of 0.82 and specificity of 0.94 for consolidation when compared to CT. Sensitivity 97 vs. 75% with CXR	Resolution of pneumonia can be tracked using TUS	Lung collapse, airway block alveolar hemorrhage
ARDS [16]	The presence of B-lines indicates an alveolar or interstitial abnormality	Interstitial edema	Sensitivity 82-92% compared to CT	Best when abnormal findings reached the pleural surface	Viral pneumonia, pulmonary embolism
Cardiogenic pulmonary edema [17,18]	Profuse bilateral B-lines with smooth pleural morphology	Pulmonary artery occlusion pressure (PAOP) estimation with TTE	Sensitivity 0.90 and Specificity 0.93 with CT. Sensitivity 95% vs. 55% with CXR	TUS that includes analysis of A- and B-lines correlates with the PAOP and may distinguish patients with cardiogenic pulmonary edema (elevated PAOP) from those with acute lung injury (normal PAOP)	Interstitial pneumonia, ARDS

The presence of pleural effusion or hepatic hydrothorax, underlying consolidation of the lung, liver abscess with rupture, pulmonary embolism, etc., are relevant, urgent situations that must be assessed in critically ill patients with liver disease. The ultrasonography machine should be positioned such that the operator can view the screen with ease, can maneuver the probe, and use a biopsy gun or needle with ease. Ambient light can be adjusted to improve the screen contrast. The clinician then adjusts the depth and gain settings for visualizing the pleura and area of interest. The probe should be held perpendicular to the skin, and the marker should be oriented in the cranial direction. The scanning plane should be adjusted such that the intercostal space (ICS) is central and the ribs frame on either side. This ensures that with an abdominal preset, a longitudinal view of the interspace is obtained, and structures near the skin point to the top of the screen, and deeper structures project to the bottom of the screen. Depth measurements and size of lesions can be measured with calipers [21].

Anatomic boundaries

**Figure 1:**
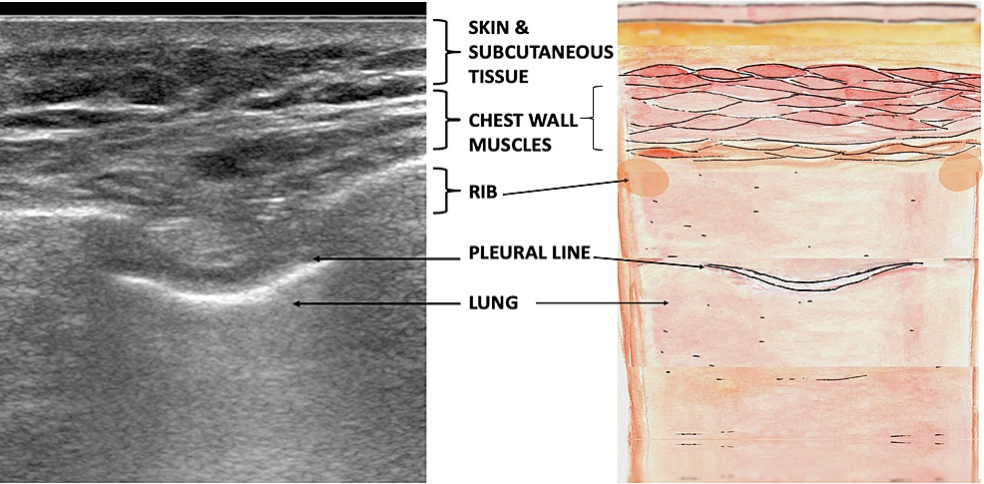
Schematic diagram showing the layers of the chest wall

The three structures identified are the diaphragm, chest wall, and lung. When placed in the intercostal space, the linear probe images from the skin inwards and shows the layers of chest wall muscle, intercostal muscle, hyperechoic pleural line, and lung (Figure 1).

**Figure 2:**
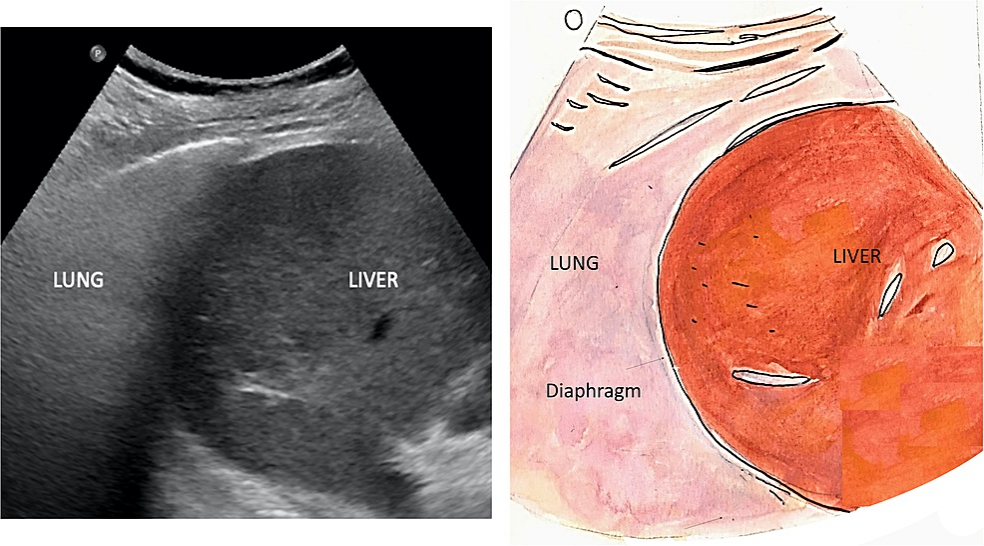
Visualization of the right lung base

**Figure 3:**
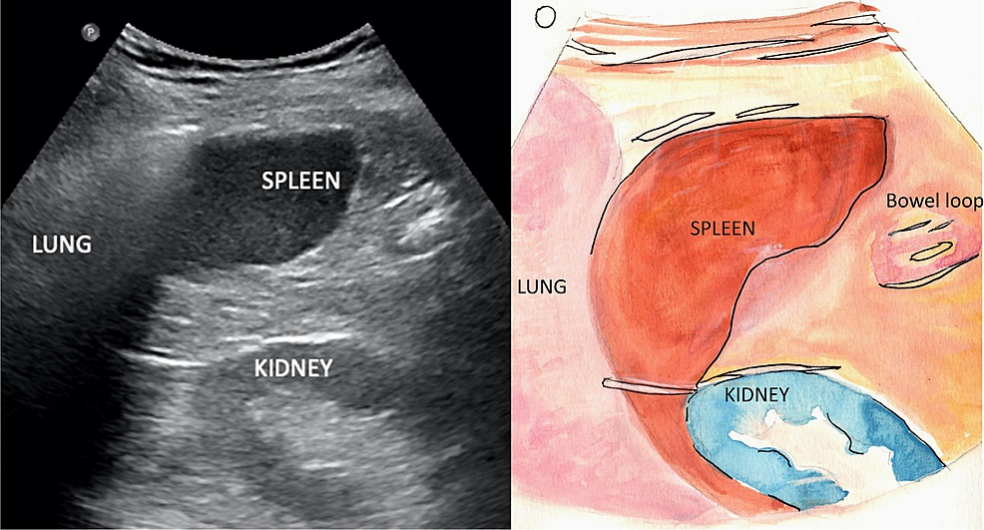
Visualization of the left lung base

The diaphragm is identified as an echogenic curvilinear structure above the liver on the right and the spleen on the left, which descends caudally with inspiration. The hepatorenal and splenorenal recesses are bounded by the liver and spleen cranially and limited by the respective kidney caudally on either side. The position of the diaphragm may be displaced by a large pleural effusion, paralysis, tense ascites, or morbid obesity [2,4]. Figures 2, 3 show the appearance of the right and left lung bases on TUS, respectively.

Lung sliding is the respiration-related movement of the pleural line seen as a shimmering mobile hyperechoic line, which is the interface of the lung with the visceral pleura and the intercostal muscle.

Lung ultrasound score

Four lung aeration patterns are defined and graded as a semiquantitative global score [18-21].

**Figure 4:**
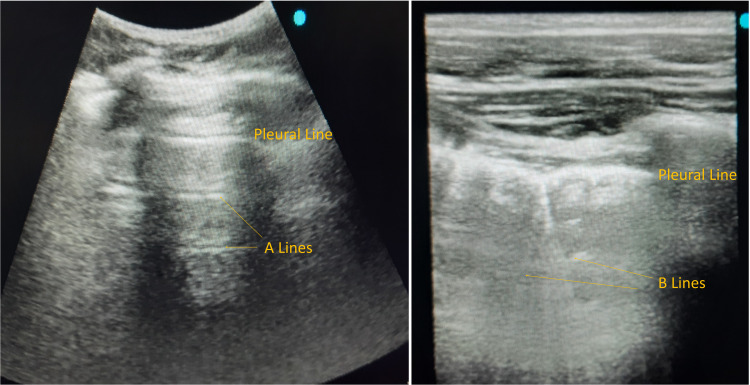
The presence of 'A' lines and 'B' lines as seen on lung ultrasound

1. Normal lung aeration: lung sliding present with A-lines and less than two isolated B-lines. A-lines represent normal lung aeration.

2. Moderate loss of lung aeration (B1 lines): multiple well-defined B-lines. B-lines represent interstitial edema (Figure 4).

3. Severe loss of lung aeration (B2 lines): multiple coalescent B-Lines

4. Lung consolidation: tissue pattern has a dynamic air bronchogram.

**Figure 5:**
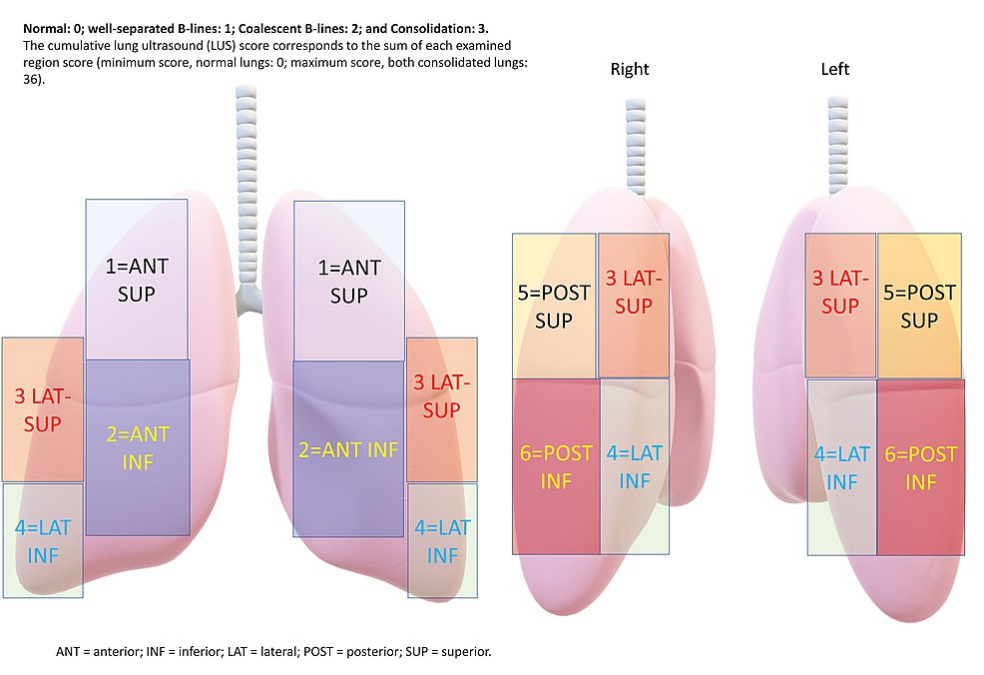
Calculation of the lung ultrasound score

Points are given as per the worst ultrasound pattern observed. Normal=0; B1 lines=1; B2 lines=2 and therefore total points are graded between 0 and 36 (Figure 5).

A higher elevated lung ultrasound score on admission is associated with a worse outcome as it suggests interstitial edema and is independently associated with a higher Acute Physiologic Assessment and Chronic Health Evaluation II (APACHE II) score and mortality [22,23]. Incorporation of LUS, whether as a scoring system or at least a protocol-based method to sequentially assess fluid status in patients with cirrhosis, is a good bedside tool to maintain fluids status or target ultrafiltration in dialysis.

Scanning technique and patient position

The scanning technique and patient position for pleural pathologies are like that for imaging of the lung. Stable patients who can sit upright should be imaged in this position, leading forward while resting their elbows on a table in front of them, exposing the back. Arms can be raised above the head to access the anterior and lateral portions of the chest wall. Patients with critical illness or those on mechanical ventilation must be imaged in the supine and semi-upright state with the ipsilateral arm adducted across the chest wall towards the opposite side. To image smaller effusions, the posterior axillary line needs to be exposed to enable a thoracentesis. This is difficult in patients who are mechanically ventilated, and a team member must be stationed to avoid displacement of the endotracheal tube [24].

Pneumothorax

The diagnosis of pneumothorax and ultrasound-guided thoracostomy tube is most useful at the bedside. The loss of pleural sliding is characteristic of pneumothorax with a few differentials. The lung point is observed at the interface between the pneumothorax (where there is no apposition of the pleura) and the partially deflated lung (where there is still apposition of the two pleural surfaces), where pleural sliding is observed. The lung point refers to the respiration phase varying, the intermittent appearance of lung sliding at a point where sliding disappears at the start of the pneumothorax area [25]. Xirouchaki et al. reported the sensitivity and specificity of LUS as 100% and 78% for consolidation, 94% and 93% for interstitial syndromes, 75% and 93% for pneumothorax, and 100% and 100% for pleural effusion [26]. The sensitivity of chest radiograph (CXR) diagnosis was lower: 38% and 89% for consolidation, 46% and 80% for interstitial syndromes, 0% and 99% for pneumothorax, and 65% and 81% for pleural effusion. Zanobetti et al. compared the concordance of TUS, chest radiography, and chest computed tomography (CT) scans in 404 mechanically ventilated patients. The performance of TUS was like chest radiography for the identification of pulmonary edema, pneumothorax, and consolidation and was superior for the identification of pleural effusion [27]. Color Doppler can be used to identify blood vessels in the pleura and can be used to avoid bleeding in pleural taps or drain insertions in patients with liver abscesses.

Pleural effusion and hepatic hydrothorax

Patients with cirrhosis frequently develop hepatic hydrothorax, and reactive pleural effusions develop in patients with liver abscesses. Another scenario is to image synpneumonic effusions in patients or post-liver biopsy bleeding. Patients with pleural tuberculosis or lung masses also develop effusions. Hepatic hydrothorax is characterized by the development of pleural effusion due to diaphragmatic defects, which suck in portal hypertensive ascites due to the negative intrapleural pressure. The majority occur in the right hemithorax. A hemothorax is defined as a bloody pleural effusion with a hematocrit exceeding half the value in peripheral blood [28]. TUS was superior to chest radiography for the diagnosis of pleural effusion (93% versus 47%), alveolar consolidation (97% versus 75%), and alveolar-interstitial syndrome (95% versus 55%) [29]. Svigals et al. reported that pleural ultrasound had a sensitivity of 69.2% (95% CI: 48.2% to 85.7%) and specificity of 90.0% (95% CI: 76.3% to 97.2%). Chest CT scan had a sensitivity of 76.9% (95% CI: 56.3% to 91.0%) and specificity of 65.0% (95% CI: 48.3% to 79.4%). CXR had a sensitivity of 61.5% (95% CI: 40.6% to 79.8%) and specificity of 60.0% (95% CI: 43.3% to 75.1%). Pleural ultrasound appears to be a superior modality to rule in a complex pleural effusion when compared with chest CT scan and CXR [30].

Use of TUS in intensive care practice in a liver ICU

The use of TUS has changed intensive care practice the world over, with increased application in hepatology and transplant practice. TUS can be applied to guide weaning protocols in patients with liver disease, reduce post-extubation stress [23] and assess the effect of antimicrobial therapy in patients with ventilator-associated pneumonia, septic shock [31], and recruitment strategies using peak end expiratory pressure (PEEP) in patients on mechanical ventilation [32]. Reduction in LUS score and improvement in consolidation, in conjunction with ventilatory requirement, serum biomarkers of sepsis-like procalcitonin, and hematological scores, can help determine the resolution of pneumonia. We routinely use the daily POCUS round in the ICU to determine the improvement in the LUS score in patients with a critical illness. When clinical parameters, fraction of inspired oxygen (FiO_2_) requirement, sepsis markers and cultures, and LUS score show an improving trend, the patient can be initiated on a de-escalation protocol. Two additional indices are of use in critically ill patients with liver disease, especially ACLF: the oxygenation index and the extravascular lung water (EVLW) index. 

Oxygenation index (OI) is a score used in intensive care practice to measure FiO2 and its use in the body. It is calculated using the formula OI= (FiO_2_ x M_PAW_)/PaO_2,_ where FiO_2_ refers to the fraction of inspired oxygen in %, M_PAW_ refers to the mean airway pressure in mmHg, and PaO_2_ refers to the partial pressure of oxygen in arterial blood, in mmHg. The higher the OI, the lower is the needed FiO_2_ to maintain a higher PaO_2_.

The EVLW index is the amount of water contained within the lung parenchyma that is outside the pulmonary vasculature. It is usually measured by specific thermodilution techniques, and the use of LUS to help estimate the EVLW is under scrutiny.

Alveolar interstitial syndrome (AIS) can be diagnosed using TUS to detect pulmonary edema, which appears as diffuse B‐lines. AIS is seen in several conditions including, but not limited to acute decompensated heart failure, noncardiogenic pulmonary edema, bilateral pneumonia/pneumonitis, and lung cancer [33,34]. Extensive B-Lines reduced functional vital capacity (FVC) and forced expiratory volume in the first second (FEV1) in a study on pre-liver transplant surgical evaluation, and they may be an independent factor in worsening pulmonary function in these patients [35,36]. Extravascular lung water (EVLW) can also be used as an end point in determining fluid status during liver transplantation or cirrhosis with shock in the intensive care unit [37-39]. The recent COVID-19 pandemic expanded the use of POCUS in liver intensive care practice. Peng et al. showed TUS could predict the clinical course and outcome of COVID-19 in an initial study from China [40]. Similar data have been reported by Lichter et al., and the LUS score has gained acceptability with shorter protocol modifications by imaging only the anterior or lateral segments [41]. Figure 5 shows patterns of pneumonia, lung collapse, and pleural effusion in patients with cirrhosis and ascites, which show difficult to interpret radiographs.

**Figure 6:**
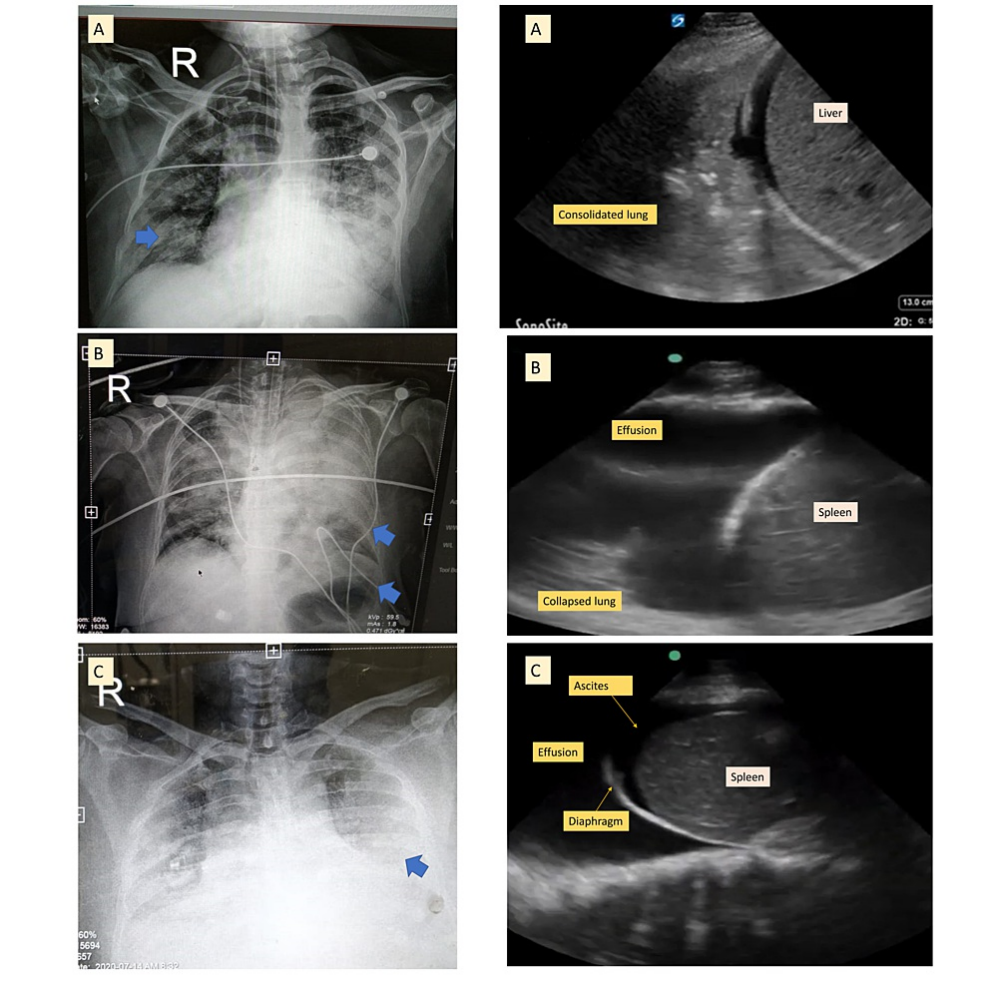
Visualization of different pneumonia patterns on chest radiograph in patients with cirrhosis and correct interpretation using lung ultrasound

TUS simplifies the diagnosis as an iterative point-of-care (POC) tool.

Ultrasound-guided procedures

A central venous access device is defined as a catheter that has its tip located in the superior vena cava, right atrium (RA), or inferior vena cava (IVC). Central venous catheters include central venous lines (jugular or subclavian), dialysis catheters, and peripherally inserted central lines. These are commonly inserted in patients with cirrhosis and ACLF for venous access or dialysis. The use of the central venous pressure (CVP) to estimate volume status in patients with hepatorenal syndrome is largely abandoned [42]. TUS has limitations as ultrasound resolution decreases with depth. Vascular structures are difficult to identify in morbidly obese patients or those with anasarca. Volume depleted patients with ACLF also have coagulation failure, which may make central venous access difficult, in addition to coagulation failure-related risk. Spencer et al. have suggested rapid central vein assessment (RaCeVA) as a systematic way for ultrasound assessment before central venous catheterization [43]. Thoracentesis and drain insertions and drainage of liver abscesses are frequently done in the ICU using similar techniques. The POCUS of the thorax and bedside echocardiography are complementary techniques in the ICU. We have previously described the method, interpretation, and utility of telecardiology tools to improve time to diagnosis in critically ill patients with liver disease [44].

Pulmonary complications of cirrhosis

The two pulmonary syndromes in cirrhosis deserve mention here as they are relevant in managing patients with cirrhosis and ACLF who are critically unwell and have a bearing on transplantation outcomes. Hepatopulmonary syndrome (HPS) is caused by impaired pulmonary capillary oxygenation due to the bypass of the alveolar circulation by intrapulmonary vascular dilations (IPVDs). It can be screened at the bedside using an intravenous agitated saline contrast POC echocardiography in tandem with arterial blood gas analysis. HPS is diagnosed if the pulmonary alveolar arterial oxygen gradient (AaO2) ≥ 15 mmHg, or ≥ 20 mmHg in persons aged >64 years [45]. Pulmonary capillaries are normally <8-15 microns. If microbubbles (>10 microns) appear in the left heart after three-to-six cardiac cycles of injecting the agitated saline in the antecubital vein, it suggests that IPVDs are present, indicating the need for confirmatory tests for HPS [46]. The second pulmonary syndrome is porto-pulmonary hypertension (POPH), diagnosed by right heart characterization, with a resting pulmonary artery pressure (mPAP) ≥ 25 mmHg. In contrast with HPS, POPH is characterized by pulmonary vessel vasoconstriction. POC echocardiography can determine the right ventricular systolic pressure (RVSP) and pulmonary arterial pressure (PAP) by continuous-wave doppler if tricuspid regurgitation (TR) is present. Systolic PAP can be measured by the peak TR velocity jet, and right atrial pressure can be determined by the simplified Bernoulli equation based on the inferior vena cava (IVC) diameter and respiratory variation [47]. Another bedside parameter to judge RA pressure is dynamic IVC measurement. An IVC diameter of < 2.1 cm that collapses > 50% with a sniff indicates normal RA pressure of 0-3 mmHg (range: 0-5 mmHg) and an IVC > 2.1 cm that collapses < 50% with a sniff or < 20% on quiet inspiration suggests a high RA pressure of 15 mmHg (range: 10-20 mmHg).

Cirrhotic cardiomyopathy

The use of TUS and POC echocardiography is now complimentary. Patients with critical illnesses like variceal bleed, hepatorenal syndrome (HRS), and sepsis frequently have underlying cirrhotic cardiomyopathy (CCM), which is seen in 30-50% of individuals. CCM may be asymptomatic and may be first diagnosed on transplant workup or may be diagnosed for the first time in case of acute stress like sepsis, bleeding, or interventions like transjugular intrahepatic portosystemic shunt [48]. These situations, which require the cirrhotic heart to increase the cardiac output, often precipitate heart failure, indicating there is a limited cardiac reserve. This has important implications in the management of critically ill patients with cirrhosis at the onset of shock, as they might not respond to a fluid challenge and require early use of vasopressors. Hence combing basic echocardiography, TUS, and IVC dynamics, we can detect pulmonary and cardiac issues in patients with liver disease, determine the dose of fluids for resuscitation, initiate timely vasopressors, and determine the dose of dialysis and safety of albumin or blood components in patients with volume overload.

Limitations of POCUS of the lung and heart

TUS has limitations related to operator and patient-dependent factors. TUS requires correct clinical skills and interpretation of LUS findings, which requires short training of operators. Easily identifiable LUS signs, as described in this review, can be taught with brief training videos, like ruling out pneumothorax or diagnosing a pleural effusion [49]. In patients with cirrhosis, the presence of ascites and coagulopathy often makes movement and guided procedures difficult. Obesity, the presence of subcutaneous emphysema, and large dressings make examination and propagation of the ultrasound beam difficult in post-operative patients and those on mechanical ventilation [50]. However, in the hands of an experienced operator, the LUS gives better real-time information in critically unwell patients as a part of the POCUS examination for multiple organ systems. LUS cannot detect lung hyperinflation due to increased thoracic pressures. Tense ascites make it difficult to find a suitable window for IVC dimension estimation, and a poor echocardiographic window makes it difficult to assess the heart. In patients on high PEEP, there is increased right ventricle (RV) pressure which causes a bulge of the RV into the left ventricle. Also, a 5 cm increase in PEEP will be associated with a 2.5 cm H2O increase in CVP [51].

## Conclusions

POCUS is increasingly used in critically ill patients with cirrhosis and liver failure for diagnosing pleural and pulmonary parenchymal pathology, guide ventilatory pressures, and diagnose extubation failures in patients on mechanical ventilation. The use of POCUS has greatly improved our diagnostic and therapeutic finesse, enabled the safe introduction of drains, evidence-based use of anticoagulation, diuretics, and antibiotics, and is increasingly accepted in daily intensive care practice in the liver intensive care and transplantation units. We could better titrate ventilatory support and guide weaning protocols, thoracentesis, drain placement, and central venous access, which are central to the management of critically ill patients with liver disease. The use of basic echocardiography to assess volume status, diagnose systolic and diastolic dysfunction, and screen liver-specific syndromes like hepatopulmonary syndromes and porto-pulmonary hypertension is of increased relevance. The combined use of POC TUS and echocardiography has greatly improved care for critically ill patients with liver disease and liver transplantation. 
